# Paid parental leave and family wellbeing in the sustainable development era

**DOI:** 10.1186/s40985-017-0067-2

**Published:** 2017-09-15

**Authors:** Jody Heymann, Aleta R. Sprague, Arijit Nandi, Alison Earle, Priya Batra, Adam Schickedanz, Paul J. Chung, Amy Raub

**Affiliations:** 1 UCLA Fielding School of Public Health, 650 Charles E Young Dr S, Los Angeles, CA 90095 USA; 2 WORLD Policy Analysis Center, UCLA Fielding School of Public Health, 621 Charles E. Young Drive S, 2213-LSB, Los Angeles, CA 90095 USA; 3 Institute for Health and Social Policy and Department of Epidemiology, McGill University, 1130 Pine Avenue West, Montreal, Montreal, H3A 1A3 Canada; 4 U.C. Riverside School of Medicine, 900 University Ave. Riverside, Riverside, CA 92507 USA; 5 Department of Pediatrics, David Geffen School of Medicine, UCLA, 10960 Wilshire Blvd., Suite 960, Los Angeles, CA 90024 USA; 6 Department of Pediatrics, David Geffen School of Medicine, UCLA, 10833 LeConte Ave, B2-433 MDCC, Los Angeles, CA 90095 USA

**Keywords:** Maternal health, Infant health, Paid leave, Breastfeeding, Sustainable development goals, Social determinants of health, Gender equality

## Abstract

**Background:**

The Sustainable development goals (SDGs) have the potential to have a significant impact on maternal and child health through their commitments both to directly addressing health services and to improving factors that form the foundation of social determinants of health. To achieve change at scale, national laws and policies have a critical role to play in implementing the SDGs’ commitments. One particular policy that could advance a range of SDGs and importantly improve maternal and infant health is paid parental leave.

**Methods:**

This article analyzes literature on paid leave and related policies relevant to SDG 1 (poverty), SDG 3 (health), SDG 5 (gender equality), SDG 8 (decent work), and SDG 10 (inequality). In addition, this article presents global data on the prevalence of policies in all 193 UN Member States.

**Results:**

A review of the literature finds that paid parental leave may support improvements across a range of SDG outcomes relevant to maternal and child health. Across national income levels, paid leave has been associated with lower infant mortality and higher rates of immunizations. In high-income countries, studies have found that paid leave increases exclusive breastfeeding and may improve women’s economic outcomes. However, factors including the duration of leave, the wage replacement rate, and whether leave is made available to both parents importantly shape the impacts of paid leave policies. While most countries now offer at least some paid maternal leave, many provide less than the 6 months recommended for exclusive breastfeeding, and only around half as many provide paternal leave.

**Conclusions:**

To accelerate progress on the SDGs’ commitments to maternal and child health, we should monitor countries’ actions on enacting or strengthening paid leave policies. Further research is needed on the duration, wage replacement rate, and availability of leave before and after birth that would best support both child and parental health outcomes and social determinants of health more broadly. In addition, further work is needed to understand the extent to which paid leave policies extend to the informal economy, where the majority of women and men in low- and middle-income countries work.

## Background

The Sustainable Development Goals (SDGs) have the potential to have a significant impact on maternal and child health, through their commitments both to directly addressing health services and to improving factors that form the foundation of social determinants of health [1]. Included among these, the SDGs commit governments to ensure healthy lives and promote well-being for all (SDG 3); to end poverty, including by implementing social protection systems (SDG 1); to achieve gender equality and empower all women and girls (SDG 5); to promote decent work for all (SDG 8); and to reduce inequality within and among countries (SDG 10). In total, the SDGs comprise 17 goals and 169 targets and are far more comprehensive with respect to social and environmental determinants of health than the 8 goals and 18 targets of the Millennium Development Goals that preceded them. The SDGs cover low-, middle-, and high-income countries.

To achieve change at scale, national laws and policies have a critical role to play in implementing the SDGs’ commitments over the coming years. Identifying approaches that have cross-cutting effects across a range of the goals would accelerate progress. Across high- and low-income countries alike, certain national policies have the potential to have impact on both the SDH and traditional health outcomes. One of these is paid parental leave, which enables women and men to take time off of work following the birth of a child while maintaining their jobs and at least partial income. Paid parental leave has the potential to have health benefits for infants and mothers, and may also reduce economic and gender inequality and improve employment conditions.

This review will evaluate the evidence for paid parental leave and assess its potential to improve a range of health and economic outcomes simultaneously. In particular, this paper will assess the impact of paid parental leave on health outcomes for children and parents, thus addressing its potential to directly affect SDG 3. In addition, this paper will examine to what extent paid parental leave advances employment and gender equality, two social determinants of health that are central to SDGs 1, 5, 8, and 10.

Finally, this paper will briefly assess the role of complementary policies, such as breastfeeding breaks and paid leave for other caregiving needs, which may support similar objectives. For each of these areas, this paper will present data illustrating the current status of each of these policies globally and outline areas for future research.

## Methodology

This article analyzes literature on paid leave and breastfeeding breaks relevant to the following Sustainable Development Goals: SDG 1 (poverty), SDG 3 (health), SDG 5 (gender equality), SDG 8 (decent work), and SDG 10 (inequality). This article builds on prior and ongoing research on parental leave in low-, middle-, and high-income countries undertaken by Jody Heymann, Alison Earle, Arijit Nandi, and colleagues from the WORLD Policy Analysis Center at the UCLA Fielding School of Public Health and the Maternal and Child Health Equity (MACHEquity) Programme at McGill University. This article also builds on a new initiative to examine all of the literature relevant to length of paid family and medical leave across the 35 countries that comprise the Organization for Economic Co-operation and Development (OECD), with deep analysis by Priya Batra, Adam Schickedanz, and Paul Chung on what duration of leave would be optimal or best support infants’ health and, separately, mothers’ health. Finally, this article draws on a systematic review conducted by Arijit Nandi and colleagues of the economic, social, and health impacts of paid parental and medical/sick leave in the OECD, which broadly examines how the availability of leave affected mothers’ and infants’ health outcomes, and whether the payment amount affected health outcomes. From these analyses, this article provides an overview of the evidence for whether paid parental leave could improve parental, child health, and economic outcomes in support of the SDGs, and further examines existing evidence and areas where further research is needed about how to shape leave policies to best support these outcomes. In addition, this article uses global policy data developed by the WORLD Policy Analysis Center and MACHEquity to illustrate how the policies examined vary worldwide. Further information on the methodology for building this data can be found at https://www.worldpolicycenter.org/methodology [2].

## Paid parental leave

### Child health and wellbeing

SDG target 3.2 calls on countries to “end preventable deaths of newborns and children under 5 years of age” by 2030 [3]. Recent research has found that longer periods of maternal leave reduce infant mortality in countries at all income levels. In a study of nearly 300,000 live births across 20 low- and middle-income countries (LMICs), using longitudinal data and multilevel models, Nandi et al. [4] found that each additional month of paid maternity leave was associated with 7.9 fewer infant deaths per 1000 live births (95% CI 3.7, 12.0), reflecting a 13% relative reduction. Reductions in infant mortality associated with increases in the duration of paid maternity leave were concentrated in the post-neonatal period. These findings are consistent with previous research from OECD countries, which has likewise found a relationship between more generous paid maternal leave policies and lower infant mortality [5–8]. For example, in a longitudinal study of 16 high-income European countries plus the United States and Japan, Tanaka (2005) found that a 10-week extension in paid maternal leave reduced infant mortality by 2.3–2.5% [7].

Two of the mechanisms by which paid leave may lower mortality are by increasing the initiation and duration of breastfeeding, widely regarded as one of the most effective downstream infant health interventions, especially in LMICs [9]; and by supporting parents’ ability to ensure their child receives essential immunizations and other post-natal care. Studies have found that more generous paid parental leave supports both these practices.

For example, in a range of studies from high-income countries, researchers have found that extending the duration of paid leave increases rates of breastfeeding for at least the critical first six months of the infant’s life [10–13]. Following reforms to Canada’s maternal (maternity plus parental) leave policy, which increased from 6 months to nearly 1 year in most provinces in 2000 [14], the share of women breastfeeding exclusively for at least 6 months increased by between 7.7 and 9.1 percentage points, according to a study that used longitudinal survey data to examine changes in health practices before and after the policy change [10]. One longitudinal cohort study from Sweden also found that when men took paternity leave, their infants were more likely to be breastfed at 2, 4, and 6 months [15], presumably through increased ability of fathers to support mothers’ breastfeeding efforts. Few studies of paid leave and breastfeeding initiation and duration have been conducted in the specific context of LMICs.

Similarly, studies from high-income countries suggest that more generous paid leave may be associated with higher rates of on-time immunizations [16, 17]. For example, Ueda et al. [17], using a logistic regression model of survey responses, found that when mothers in Japan took parental leave, their children were significantly less likely to be behind schedule on immunizations at 36 months. In a longitudinal study of OECD countries, however, Tanaka (2005) found no statistically significant relationship between the availability of maternal leave, looking separately at both job-protected paid leave and unpaid leave, and immunization rates within the first year [7]. The author hypothesized that the lack of a statistically significant relationship could be due to the relatively high vaccination rate for diphtheria, tetanus, and pertussis (DTP) across these countries, which has remained stable over time, in addition to many countries’ practice of delaying the measles immunization until 12 months. This also could be due to the amount and nature of leave changes over the time period studied.

More recently, studies have found a positive relationship between paid maternal leave and vaccination rates in LMICs [18, 19]. In a study of around 250,000 live births across 20 LMICs, using longitudinal data and multilevel models, Hajizadeh et al. [19] found that each additional week of paid maternity leave increased the probability of the DTP1, 2 and 3 vaccinations by 1.38, 1.62 and 2.17 percentage points, respectively. By contrast, the study did not find a significant relationship between increases in paid maternity leave and the probability of children receiving the BCG vaccine, which is typically administered right after birth while infants are still hospitalized. The findings suggest that paid parental leave can improve immunization rates in LMICs, but that the effect depends on the duration of available leave and is most pronounced for vaccines that are typically administered in series over the first several months.

Finally, one longitudinal study, applying a difference-in-differences estimate to data from five US states, found that the provision of short-term disability leave that could be taken before or after childbirth reduced the incidence of low birth weight, which the author posited was likely driven by higher take-up of antenatal care, though data on the timing of leave-taking was unavailable to confirm this theory [20]. Some research also suggests that there are benefits for children’s cognitive development when their parents take paid leave around the time of their birth [21, 22]. For example, in a quasi-experimental study of Norway, contrasting births before and after the introduction of a paternity leave quota, Cools et al. [22] found that children’s performance in school at age 16 increases when their father has access to a policy incentive to take paternity leave, especially in families where the father has more education than the mother. In addition, at least one study has found that paid parental leave may reduce the incidence of pediatric hospital admissions due to child abuse [23].

## Earnings and employment

Decent employment and safe working conditions are foundational to health [24], and also central to SDG 8. SDG 8.5 calls for “achiev[ing] full and productive employment and decent work for all women and men,” while SDG 8.8 outlines states’ responsibility to “[p]rotect labour rights and promote safe and secure working environments for all workers,” including those in precarious employment [3].

Consistent employment with an adequate wage often provides the most sustainable pathway out of poverty, and enables families to better meet their basic needs. Maternal employment in particular has intergenerational benefits for health and well-being; when women have their own earnings and assets, investments in children’s health and education increase [25, 26]. SDG targets 1.1 and 1.2 call for eradicating extreme poverty and otherwise reducing the share of people in poverty by half, while SDGs 5.1, 5.7, 5.9, and 10.2 all support strengthening women’s access to employment and economic opportunities [3].

In a range of studies, paid leave has been associated with increases in both women’s earnings and their long-term attachment to the labor force [5, 27–29]. For example, Waldfogel (1998) found that the availability of maternity leave was associated with increases in wages for up to 5 years among mothers who returned to work at some point after childbirth in Britain and the USA, while Rasmussen (2010) found that introducing 6 weeks of paid parental leave in Denmark, which supplemented the 14 weeks of post-birth leave already available to mothers, had small positive effects on mothers’ earnings, work experience, and employment rates in the 5 years after the reform [27, 28]. Similarly, two studies from California, both using a difference-in-differences approach, found that the state’s paid family leave policy increased working mothers’ take-up of leave, and was associated with increases in the work hours and wages of mothers with young children [30, 31]. Over time, ensuring broad and inclusive participation in the workforce supports economic growth and higher GDPs, which is the aim of SDG 8.1 [3]. For example, in a 2012 study, researchers found that eliminating the gender gap in labor force participation in OECD countries could result in a 12% increase in GDP across the OECD as a whole by 2030 [32]. In the USA, the only OECD member that lacks paid maternal leave, economists have estimated that the lack of “family friendly” policies is responsible for nearly a third of the relative decline in female labor force participation in the USA compared to other OECD countries between 1990 and 2010 [33]. These findings suggest that the provision of leave may have significant benefits for the economy that should be considered alongside any assessment of its costs.

However, one important question is what the ideal duration of paid leave is to support women’s economic outcomes. The International Labor Organization (ILO) Maternity Protection Convention (2000) established a minimum global standard of 14 weeks of paid maternal leave for working mothers [34]. In its supplemental Maternity Protection Recommendation, the ILO recommended a minimum of 18 weeks [35]. Notably, both of these fall short of the 26 weeks that would fully support the 6 months of exclusive breastfeeding recommended by WHO. As of 2014, 53% of countries met the ILO Convention standard, including 47% of low-income countries, 43% of middle-income countries, and 77% of high-income countries, showing that it is feasible for countries across economic levels to provide paid leave [36–39]. Twenty-six percent of countries, including 6% of low-income countries, 19% of middle-income countries, and 52% of high-income countries, provide at least 26 weeks, facilitating mothers’ ability to meet the WHO breastfeeding guidelines [36]. Finally, 17% of countries, including 3% of low-income countries, 15% of middle-income countries, and 30% of high-income countries, provide a year of paid leave or more [36]. In addition, 86% of countries provide a maximum wage replacement rate of at least two-thirds regular wages, as recommended by the ILO, which is another important factor in determining whether women can afford to take leave and how leave will shape economic outcomes [36].

A study of 21 high-income countries, using multilevel models to estimate women’s earnings as a share of their family’s income, found that access to more than 24 weeks of paid leave increased women’s long-term relative contribution to household income [40]. Yet other research has found that longer leaves, such as those lasting more than 9 months or more than 1 year, may have negative effects on women’s earnings, though approaches to measuring these effects have varied across studies [41–44]. For example, Schönberg and Ludsteck (2014) compared earnings of mothers who took leave 3 months before a series of increases in leave duration in Germany with earnings of mothers who took leave 3 months after [44]. Following a reform that increased maternal leave from 6 to 22 months, up to 4% of mothers decided not to return to work, while mothers’ labor market income fell by approximately 8% 6 years after birth, and employed mothers’ earnings fell by approximately 3% for 2 to 6 years after birth [44].

Further understanding the mechanisms and tipping points for this variation in outcomes for longer leaves will be important for determining the optimal length of leave to support women’s earnings and employment.

In addition, if paid leave is only available to women, it may lead to greater gender discrimination in the labor market. This discrimination could in turn widen the existing gender gaps in wages and employment, partially offsetting some of the benefits of paid leave to women’s earnings. The following section examines the evidence for providing adequate leave to both parents in more detail.

### Gender equality

Gender inequality has deep impacts on health, whether through shaping the allocation of wealth and resources, determining whose health needs are recognized and prioritized, or directly affecting physical and mental health as a result of increased exposure to violence and discrimination. Gender inequality also has repercussions specifically for maternal and child health, and gender bias has been identified as a contributor to inadequate access to prenatal care and nutrition during pregnancy, lower birth weights, higher rates of maternal mortality, and undernourishment of female children [45, 46]. While the consequences of gender inequality and traditional gender norms primarily disadvantage women, men also feel the effects, which manifest in fewer opportunities to participate in caregiving, increased risk-taking, and shorter life expectancies [47]. Against this backdrop, SDG 5.4 calls on countries to promote “shared responsibility within the household and the family” [3].

**Fig. 1:**
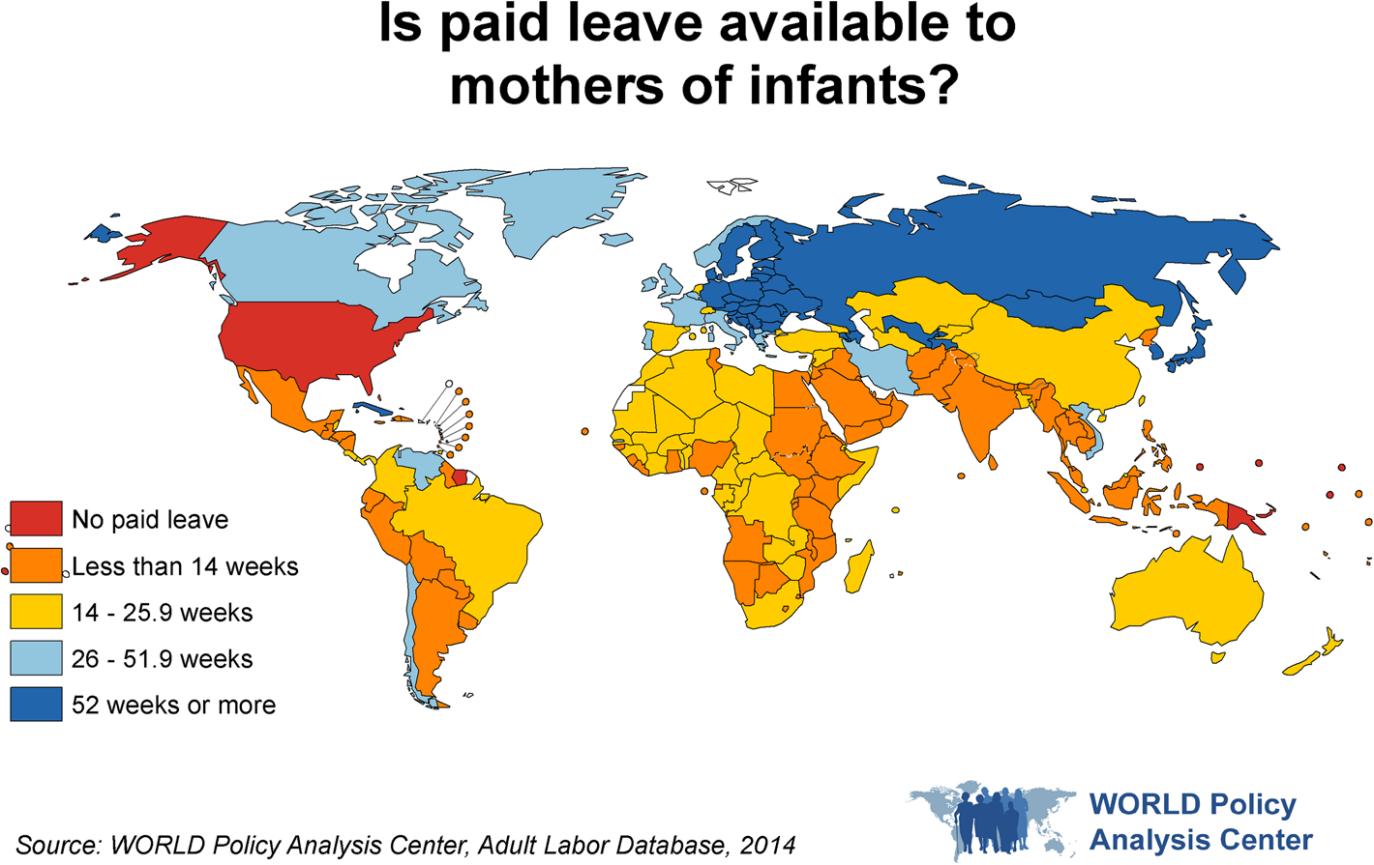
Is paid leave available to mothers of infants?

Research suggests that the structure of labor policies like paid leave has significant implications for gender equality, making these policies a potentially important lever for accelerating progress toward all of SDG 5’s targets, as well as SDGs 10.2, 10.3, and 10.4 [3]. For example, a wide range of studies have found that fathers who take paid leave are more involved in childcare both during the leave period and later in the child’s life [48–50]. This evidence supports the idea that when available to both parents, paid parental leave can support gender equality at home and at work. By contrast, when paid leave is available only to women, it may reinforce the idea that women are primarily responsible for caregiving, while men are the primary earners. Currently, while all but eight countries globally provide paid leave to women, only 49% make any leave available to fathers [36] (Fig. 1). At the same time, leave for fathers tends to be for a far shorter duration; 49% of countries that make leave available to fathers provide less than 3 weeks [36].

Further, studies have shown that simply making parental leave available to men is often insufficient to increase men’s take-up. This is partly due to stigma and longstanding gender norms [51, 52], though it may also result as a consequence of the gender wage gap. If paid leave is only provided at partial wages, it makes sense for the lower-earning parent, who remains more likely in most countries to be the mother, to take a greater share of the available leave [53]. Based on data from a wide range of high-income countries from 2000 to 2013 [54], a wage replacement rate of at least two-thirds appears to be the minimum for even modest take-up by fathers, while a rate of 80–100% of regular wages is needed for broader participation. Still, even in the face of the gender imbalance in take-up, it is important to note there is substantial data that wage replacement rates also influence whether and for how long women take leave. In the USA, which only provides unpaid leave, women are nearly twice as likely as men to report that they needed leave but were unable to take it, while nearly half of those with unmet need for leave cite lack of affordability as the key reason [55]. Likewise, in states that provide paid leave, including Rhode Island and California, leave uptake was limited for both women and men due to the low wage replacement rates, which provide a maximum of 60 and 55% of wages, respectively [56, 57].

**Fig. 2:**
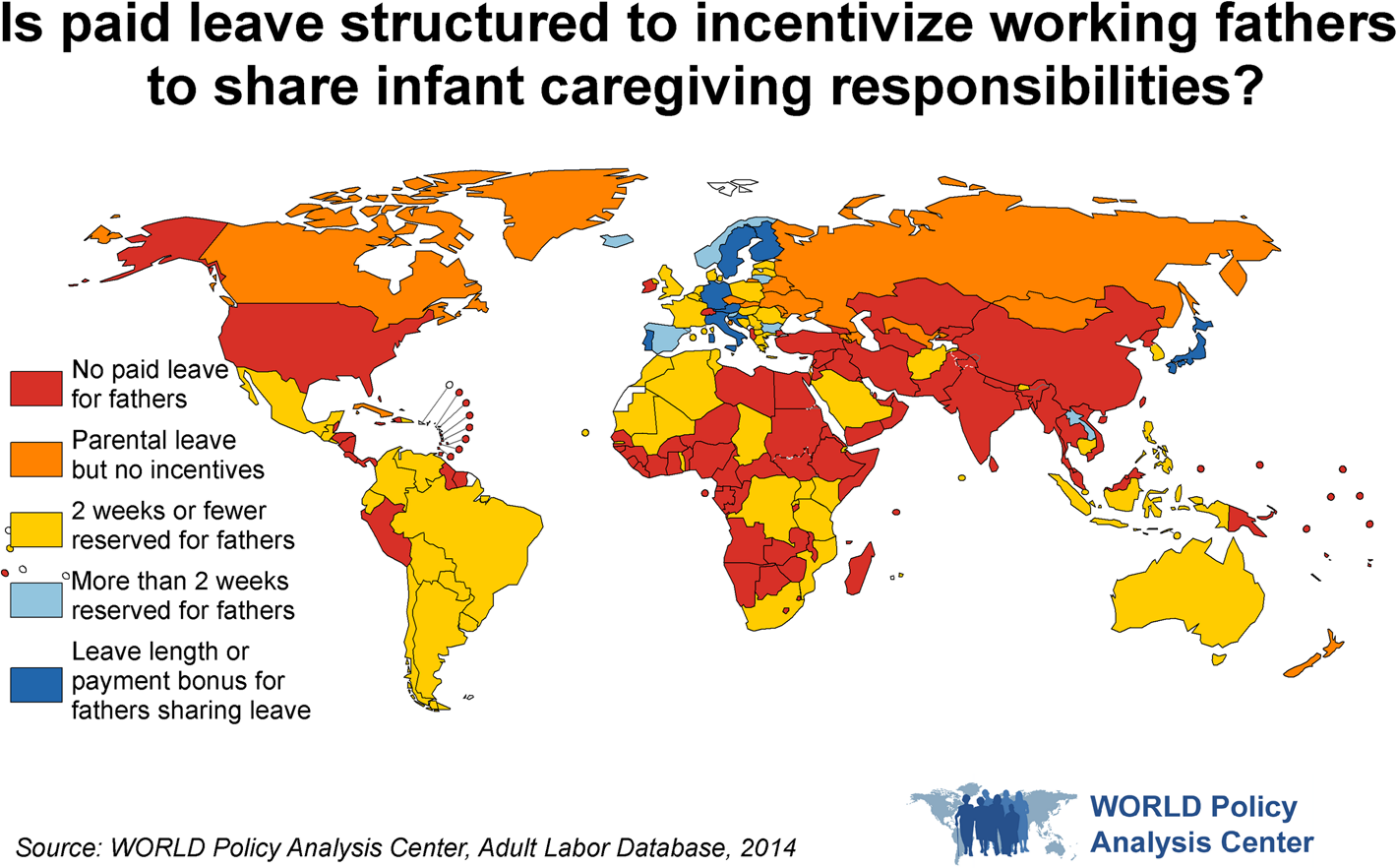
Is paid leave structured to incentivize working fathers to share infant caregiving responsibilities?

In addition to ensuring wage replacement rates are high enough for families to be able to afford for both parents to take leave, two policy approaches, “use-it-or-lose-it” and “bonus” leave, have been effective in encouraging men to take leave. Research across the OECD has shown that reserving leave for fathers through “use-it-or-lose-it” schemes has markedly increased the share of fathers taking leave [58–60]. For example, in Korea, three times as many men took leave following the introduction of 1 year of non-transferable parental leave as an individual entitlement for each employee in 2007 [61]. These policies may also reduce stigma for leave-taking, which is sometimes seen as a signal of low commitment at work. Similarly, economic incentives or bonuses that are only available if both parents take leave have been used to increase fathers’ leave taking [53, 54]. As of 2014, however, only 15 countries reserve more than 2 weeks of leave for fathers or provide incentives for fathers to take leave [36] (Fig. 2). More research is needed in low- and middle-income countries to assess which policies would best support an increasingly equal role by fathers.

### Maternal health and wellbeing

While the research is derived primarily from higher-income countries, a range of studies have found important health benefits for women who have access to paid parental leave.

Most fundamentally, paid leave enables women to physically recover from childbirth before returning to work [62, 63]. In addition, some research suggests that paid parental leave also has benefits for mothers’ mental and emotional health, a priority under SDG target 3.4 [3]. For example, several studies have found that the availability of leave reduces the risk of postpartum depression [64, 65] while Avendano et al. [66], using a difference-in-differences approach, found that more generous maternity leave policies are associated with lower rates of maternal depression in older age. However, across both physical and mental health, length of leave may make a critical difference. According to two studies, it is after 12 weeks of post-partum leave that mothers’ self-reported measures of vitality and physical health typically begin to improve [62, 63].

Beyond these broad effects on recovery, paid postpartum leave’s facilitation of breastfeeding has specific maternal health benefits as well as benefits for the health of their infants. For example, Geller et al. [67] found that breastfeeding may reduce the risk of post-partum hemorrhage—a leading cause of maternal mortality, particularly in LMICs; SDG 3.1 calls for further reducing the global maternal mortality ratio to below 70 per 100,000 live births [3]. Likewise, Ip et al. [68], in a study of high-income countries, found that breastfeeding reduces the risk of premenopausal breast cancer and may also lower the risk of ovarian cancer.

Finally, at least one study has found an association between paid parental leave and reduced intimate partner violence [69], a critical issue highlighted in SDG 5.2 [3].

In sum, although further research is needed to confirm the effects of paid leave on maternal health, including what balance of antenatal and postpartum leave is optimal for supporting mothers’ health outcomes, existing studies suggest that it could have positive effects on recovery from childbirth and overall well-being.

### Paternal health and wellbeing

While a growing body of evidence suggests that paid leave can support women’s health, the potential benefits for men are less well studied.

Although few studies have examined this question directly, several have found that the availability of paid leave for fathers increases their involvement with their infants, which may result in greater satisfaction in their relationships with their children [48, 50]. For example, in an earlier review of the literature, primarily from the Nordic countries, O’Brien (2009) found that paid paternal leave “has the potential to boost fathers’ practical and emotional investment in infant care,” but called for greater research to understand the underlying mechanisms [50]. In a more recent study from Bangladesh, which does not currently have paid paternal leave, researchers found that fathers who arranged to take time off around the birth of a child were more involved with their children and spent more one-on-one time with them [70].

### Breastfeeding breaks

As previously noted, breastfeeding is widely considered to be a highly impactful infant health intervention. A child who is exclusively breastfed is 14 times less likely to die within the first 6 months than a child who is not breastfed, due in part to dramatic reductions in malnutrition [71, 72] and simultaneously to reductions in infections. Breastfeeding has also been associated with improved health outcomes in childhood, including improvements in neurocognitive development and lower rates of chronic diseases like diabetes (SDG 3.4) [3, 21, 73, 74]. The World Health Organization recommends 6 months of exclusive breastfeeding for infants to receive the maximum health benefits.

For mothers who want or need to return to work within this time period, paid breastfeeding breaks can facilitate the continuation of exclusive breastfeeding for the full 6 months [75]. In this way, breastfeeding breaks can serve as an important complementary policy to paid parental leave [76]. Further, while countries at all income levels have managed to afford at least some amount of paid leave, breastfeeding breaks are a less expensive policy option that can supplement shorter leave periods in some of the lower-resource countries that cannot yet afford to provide leave of longer duration.

Relatively few studies have examined the specific impacts on health of breastfeeding breaks, though the existing research suggests a positive relationship between supportive breastfeeding policies and breastfeeding rates. For example, one study from the USA found that states that had enacted breastfeeding legislation reported higher rates of exclusive breastfeeding than those without legislation [77].

**Fig. 3:**
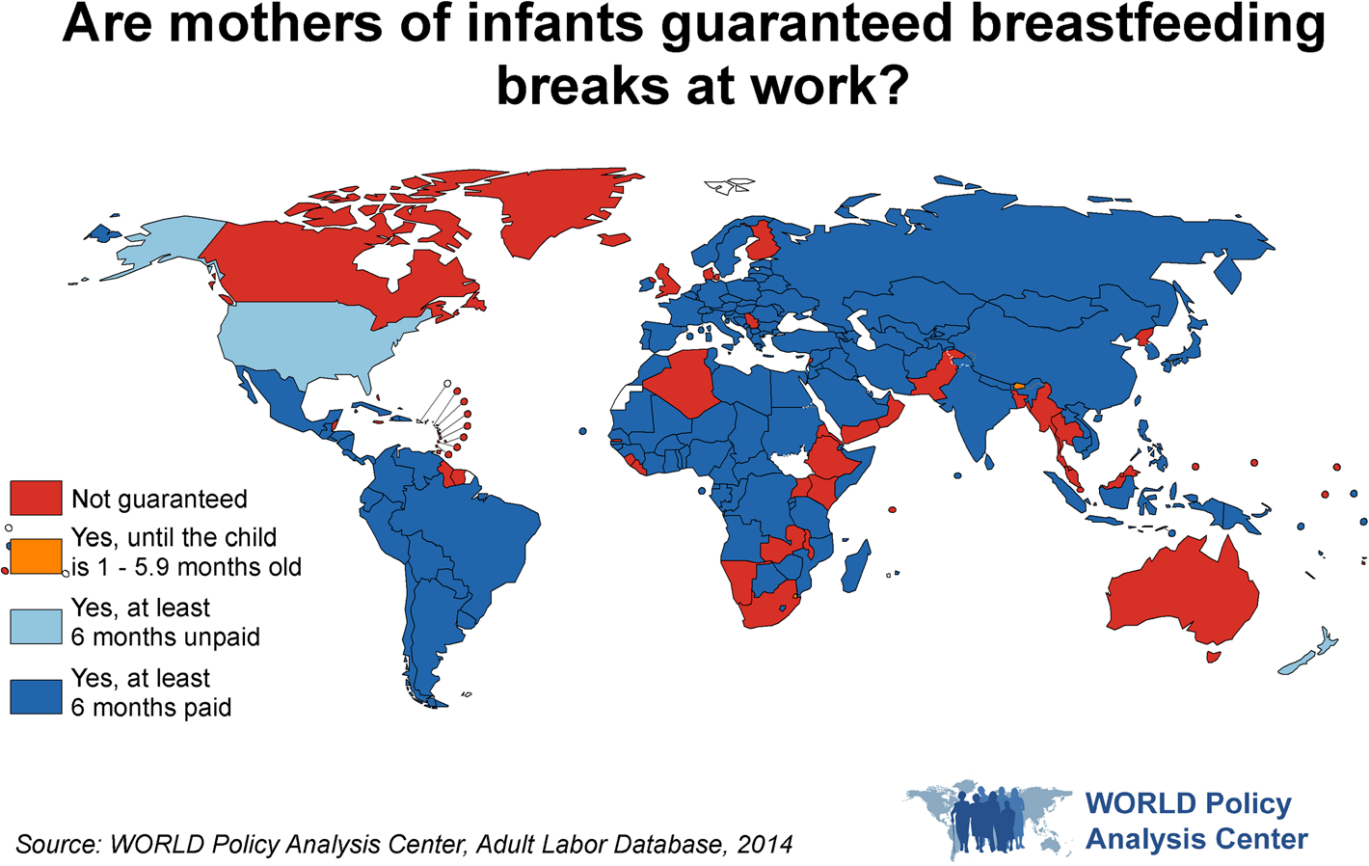
Are mothers of infants guaranteed breastfeeding breaks at work?

Further research suggests these effects may extend more broadly. In a cross-sectional study of 182 countries’ breastfeeding break policies, Heymann et al. [78] found that the guarantee of paid breastfeeding breaks until the infant is at least 6 months of age was associated with rates of exclusive breastfeeding that were 8.9 percentage points higher for infants under 6 months of age. Like paid leave, by successfully facilitating breastfeeding, legislated paid breaks are likely to have benefits for both infant and maternal health, making breastfeeding breaks another important policy area for consideration in efforts to advance the SDGs and maternal and child health more generally. As of 2014, 71% of countries guaranteed paid breastfeeding breaks until the infant was at least 6 months old, including 69% of low-income countries, 73% of middle-income countries, and 70% of high-income countries [36] (Fig. 3). More broadly, examining other labor policies that support breastfeeding, including policies around a physical space to breastfeed or pump, storage for breastmilk, and nearby infant care, would provide a more comprehensive understanding of countries’ efforts to facilitate exclusive breastfeeding by working mothers.

### Paid leave for family health needs

Caregiving needs and responsibilities extend beyond infancy. As a result, many workers have to take time off from work to care for the acute or chronic health needs of children or adult family members.

As with breastfeeding breaks, few studies have examined the specific impacts of paid leave for other family health needs. However, Earle and Heymann (2012), in interviews in the USA, found that workers who have a child with a health problem are 30% less likely to lose wages if they have access to paid leave for family health needs [79]. The study also found that female employees were 69% more likely to report losing income or wages due to caregiving, further suggesting that paid leave for all types of caregiving needs is important for both health and for gender equality. In addition, prior research has found that children recover from illness and injury faster when their parents are able to provide care [80–82], and that parents’ presence reduces emotional distress among ill children [83–86].

**Fig. 4:**
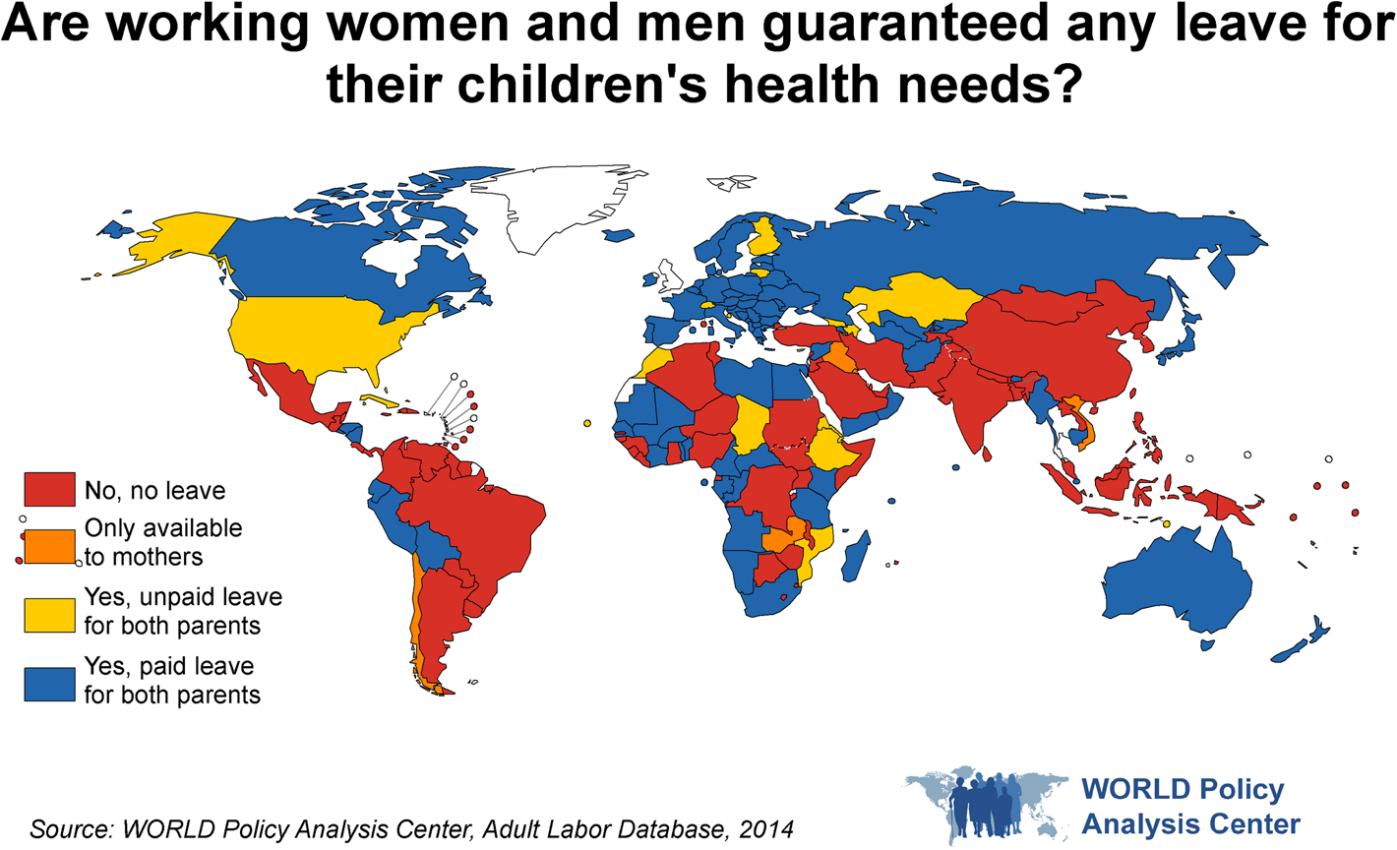
Are working women and men guaranteed any leave for their children’s health needs?

As of 2014, 45% of countries provide some form of paid leave that parents can take to meet the health needs of their children beyond infancy, while 10% provide unpaid leave for these purposes [36]. In 3% of countries, leave to care for children beyond infancy is only available to mothers [36] (Fig. 4).

**Fig. 5:**
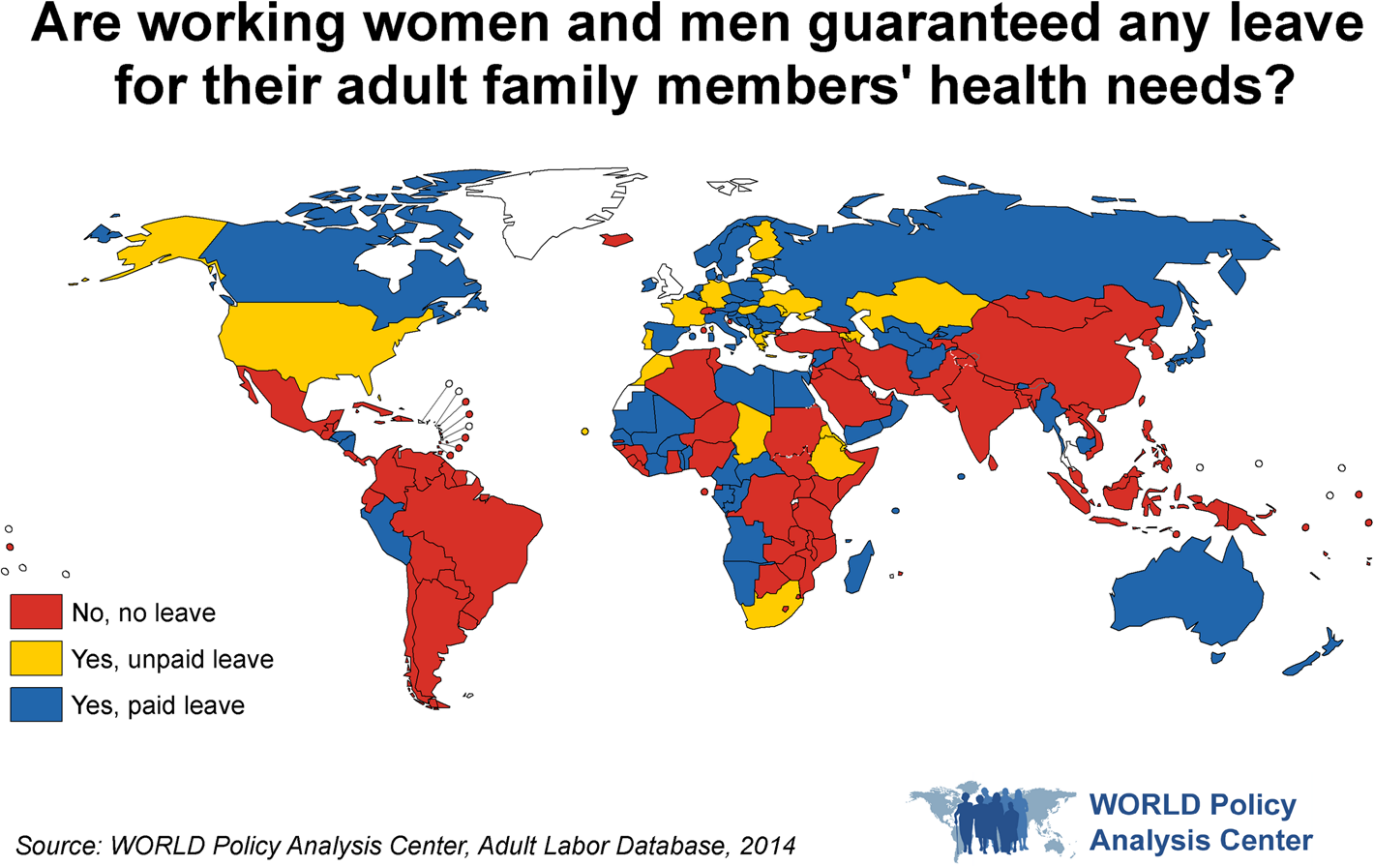
Are working women and men guaranteed any leave for their adult family members’ health needs?

Thirty-six percent of countries provide some form of paid leave that can be used to provide care to adult family members, while 13% provide unpaid leave for these purposes [36] (Fig. 5).

## Conclusions

A review of the literature reveals that paid parental leave can make an important difference across a range of SDG outcomes (SDG 1, 3, 5, 8, 10) in low- and high-income countries alike, including direct indicators of health and social determinants of health. Moreover, by supporting workforce attachment and its positive impact on long-term incomes, paid parental leave can help both national economies and family incomes [5, 30–33]. This makes paid parental leave, which already has a strong basis of support in international agreements [87, 88], an especially important policy to support realization of the SDGs across all income levels. While most countries now offer at least some paid maternal leave, many countries need to go further to ensure at least 6 months of paid leave to support exclusive breastfeeding, particularly for women in low wage jobs who may not have access to refrigeration at work or nearby childcare to make breastfeeding breaks an effective option. The world has much further to go in supporting leave for fathers, which has independent benefits for the health and wellbeing of families. Few countries reserve even a modest amount of leave for dads or have incentives for them to take leave to support equal parenting and more equal opportunities for women at work. For both parents, an adequate wage replacement rate is important to support take-up, and may be especially critical for ensuring fathers take the leave for which they are eligible, particularly given the many settings where persistent wage disparities mean men are more often the higher earner in the family. Leave for other health needs can also make a significant difference for health outcomes.

Further research is needed to understand the implications of different paid leave structures in the context of an evolving global economy, which is marked by an increase in informal employment, a reduction in average job tenure, and shifts in the average number of employees per workplace. In LMICs, the majority of parents work in informal employment, as do an increasing share of workers in high-income countries. While affecting all, this disproportionately affects women in LMICs. Preliminary data collected by the WORLD Policy Analysis Center indicates that some countries explicitly include workers in largely informal sectors like agriculture and domestic work in their paid leave policies, though others explicitly exclude them. Generally, designing benefits like paid leave as social insurance that is available to everyone in the country, rather than benefits linked to certain types of employment, can ensure they reach the full population, including often the lowest income and most marginalized workers. Regarding tenure, a preliminary WORLD analysis of OECD countries found that around one in four required a parent to have been with the same employer for 6–12 months before they would be eligible for leave; with average tenure on the decline, these types of design decisions will become increasingly consequential. Moreover, to evaluate whether all these policies are fully reaching the intended beneficiaries, further research is needed on implementation and enforcement.

Finally, to accelerate progress on the SDGs’ commitments to maternal and child health, we should monitor countries’ actions on enacting or strengthening these policies. As was the case in MDG monitoring efforts, the formal SDG monitoring process focuses almost exclusively on outcome indicators; of the 232 indicators across the 17 goals, fewer than 20 explicitly call for collecting data on national laws and policies [89]. While measuring how outcomes improve is a crucial measure of success, collecting policy data enables us to understand and recognize in real time the steps countries are taking to improve those outcomes [90]. In addition, by merging global policy data with household survey data on outcomes, as exemplified by several of the studies examined in this review, we can better understand the effectiveness of specific policy approaches across regions and socioeconomic contexts.
